# The treatment of tricuspid regurgitation

**DOI:** 10.1093/eurheartjsupp/suag053

**Published:** 2026-03-19

**Authors:** Jeroen J Bax

**Affiliations:** Department of Cardiology, Leiden University Medical Center, Leiden, The Netherlands

**Keywords:** tricuspid regurgitation, imaging, right ventricle

In this issue of the *European Heart Journal Supplement*, the thematic focus is dedicated to tricuspid regurgitation (TR): diagnosis, prognosis, and treatment. The Supplement comprises eight articles, each addressing a critical component of this increasingly relevant valvular disease.

**Chapter 1**—Zamorano and colleagues outline the clinical imperative for treating patients with significant TR. They emphasize that outcomes are poor when TR is left unaddressed; however, early recognition and timely intervention can prevent progressive myocardial damage, particularly right ventricular dilation and dysfunction.

**Chapter 2**—Donal and co-authors highlight the long-standing underdiagnosis and undertreatment of TR. With the emergence of the TRILUMINATE Pivotal and Tri-FR trials—and the supporting economic models—there is now compelling evidence that correcting TR using T-TEER systems can improve patient outcomes. The authors explore both the clinical rationale and the economic case for treating TR as a modifiable, actionable condition.

**Chapter 3**—Nickenig and colleagues present advances in pre- and peri-procedural imaging. Their chapter underscores the essential role of contemporary multimodality imaging in supporting procedural planning and success for TR interventions.

**Chapter 4**—Benfari and colleagues provide an extensive discussion on the phenotypic spectrum of TR. They highlight that significant functional (secondary) TR accounts for ∼85% of clinically meaningful cases and can be further categorized into atrial or ventricular secondary TR, each with distinct pathophysiological and imaging profiles.

**Chapter 5**—Adamo and co-authors analyse the aetiology, prevalence, and prognosis of TR in detail, offering a comprehensive understanding of disease burden and trajectory.

**Chapter 6**—Estevez-Loureiro and colleagues deliver a complete overview of the current landscape of devices available for transcatheter tricuspid valve treatment, reflecting the rapid technological progress in this field.

**Chapter 7**—Möllmann and co-authors examine the evolution of tricuspid therapies and review data from key European registries, including TRIVALVE, EuroTR, and bRIGHT. These registries offer valuable insights into the demographic and clinical characteristics of patients treated across diverse real-world European healthcare settings.

**Chapter 8**—In the concluding chapter, Adamo and colleagues present a practical update on the 2025 ESC/EACTS valvular heart disease guidelines, focusing particularly on recent evolutions in the management of mitral and TR.

In closing, we extend our thanks to all contributing authors for their thoughtful, thorough, and impactful analyses. This supplement represents the first dedicated issue centred on the diagnosis, prognosis, and treatment of TR—a condition that has rightfully gained significant attention over the past decade.

## Data Availability

Not applicable, since this article is not an original study; but a point-of-view / review.

